# Post-traumatic Patellar Tendon Repair with Ipsilateral Peroneus Tendon Augmentation Post Total Knee Arthroplasty: A Case Report

**DOI:** 10.5704/MOJ.2603.018

**Published:** 2026-03

**Authors:** S Bhattacharjee, A Prasad, A Ahlawat

**Affiliations:** 1 Robotic Joint Replacement Unit, Max Super Speciality Hospital, Saket, India; 2 Department of Orthopaedics, Sarvodaya Hospital, Faridabad, India

**Keywords:** patellar tendon rupture, total knee arthroplasty, knee trauma, medial collateral ligament, ipsilateral peroneus tendon

## Abstract

Patellar tendon rupture is an uncommon but serious complication that results in loss of knee extension during and after total knee arthroplasty (TKA), significantly impacting the patient’s quality of life. Various surgical treatments, ranging from initial repair to reconstruction, are available and accessible. In recent years, the peroneus longus tendon autograft has been utilised to restore the knee extensor system. The purpose of this case report was to present the case of a patient who had a traumatic patellar tendon rupture following TKA and requiring surgery along with peroneus tendon augmentation. A 71-year-old woman underwent bilateral robotic-assisted cruciate retaining TKA for a Grade IV arthritic knee. Post surgery, on day five patient had a history of a fall at home, following which she was unable to extend her knees. On evaluation through ultrasonography and radiographs, she was found to have a ruptured patellar tendon and sprain of the medial collateral ligament. Primary repair of the tendon along with augmentation with the peroneus tendon was performed, and the patient was followed for 12 months, at the end of which, the patient was able to achieve a good functional outcome. In conclusion, early results from patellar tendon reconstruction using an ipsilateral peroneus longus tendon autograft following TKA suggest that this technique is effective for managing acute post-traumatic patellar tendon rupture. It facilitates early recovery, yields favourable outcomes, and may reduce the risk of infection.

## Introduction

Patellar tendon rupture is a rare yet serious complication after total knee arthroplasty (TKA), with a 0.17% occurrence rate. It's a major cause of knee extensor dysfunction and can severely affect patient outcomes and quality of life^1^. Several factors may contribute to patellar tendon rupture. Tendon quality is impacted by non-injury variables such as systemic disorders (such as diabetes, steroid use, and inflammatory conditions). Traumatic ruptures are mostly caused by knee trauma, which is frequently brought on by eccentric loading. Additionally, component malposition or technical errors during surgery may lead to tendon rupture^1^.

We report a case involving a Grade II sprain of the medial collateral ligament (MCL) on the right side and a post-traumatic rupture of the patellar tendon on the left. The MCL sprain was managed conservatively, while the patellar tendon rupture was treated surgically with reconstruction and augmentation using the ipsilateral peroneus longus tendon. The patient achieved excellent results at the six-month follow-up.

## Case Report

A 71-year-old female patient received bilateral robotic-assisted cruciate retaining TKA under spinal anaesthesia using a subvastus approach for Grade IV osteoarthritis knee. Patelloplasty was performed during surgery. The patient's uncomplicated post-operative recovery allowed her to walk without assistance on day one and discharge on day three. On post-operative day five, she slipped in the bathroom, resulting in bilateral knee pain and swelling. During a clinical examination, the patient showed tenderness around the medial joint line on the right side and the anterior aspect of the tibial tuberosity and knee joint on the left side with extensor lag.

**Fig. 1:**
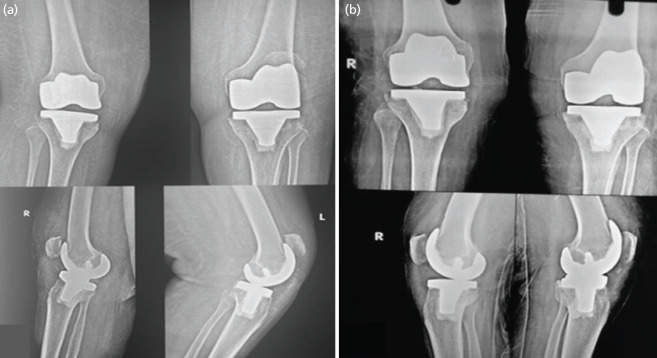
(a) Pre-operative radiograph suggestive of patella alta on the left side and medial joint line opening on the right knee, (b) post-operative radiograph.

The radiographs revealed patella alta on the left side and medial joint line opening on the right knee (Fig. 1a). An ultrasound of the left knee joint revealed a ruptured patellar tendon and a sprain of the medial collateral ligament on the right side.

**Fig. 2:**
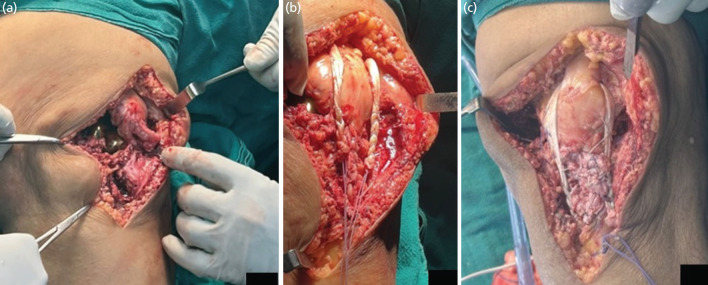
Intra-operative findings showing, (a) patellar tendon rupture, (b) patellar tendon reconstruction, (c) augmentation with peroneus tendon.

**Fig. 3:**
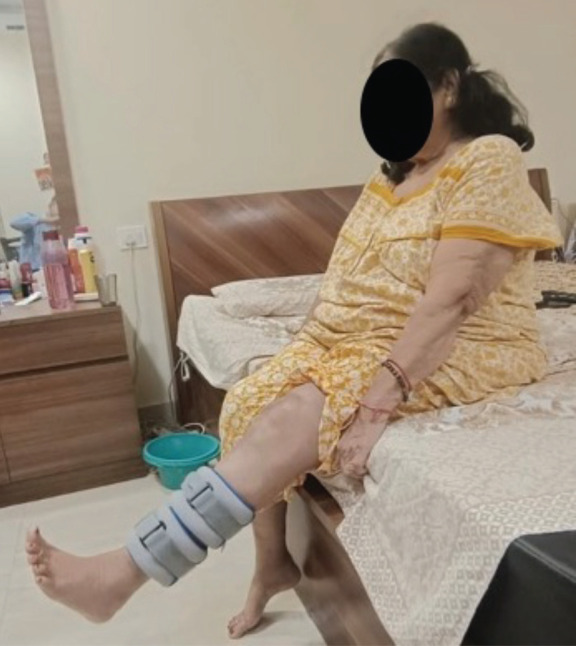
Post-operative clinical photos showing no extensor lag in left knee.

Surgical intervention involved reopening the previous incision and performing a deep dissection. Intra-operatively, a mid-substance patellar tendon rupture was observed in the left knee, following which a patellar tendon repair with peroneus tendon augmentation was performed (Fig. 2a). Peroneus longus tendon was extracted from the ipsilateral lower limb and prepared for utilisation. It was fixed using a non-absorbable suture after tensioning at 30° of knee flexion, passing under the quadriceps tendon at the superior pole of the patella and the free end of the tendon via the trans-osseous tibial tunnel (Fig. 2b and 2c). Layered wound closure was completed following haemostasis. Postoperative radiographs showed excellent restoration of patellar height (Fig. 1b). The patient's knee was immobilised with a long knee brace for four weeks before being permitted to bear full weight. Patients' knee mobilisation began four weeks after surgery with dynamic quadriceps exercises. After 12 weeks, the patient had pain-free mobility from 0° to 110° with no extension lag (Fig. 3).

## Discussion

Patellar tendon rupture is a recognised cause of extensor mechanism disruption in TKA, occurring intra-operatively or post-traumatically. Treatment options include immobilisation, direct primary repair using sutures, staples, or wire, and using biologic/synthetic grafts or allograft reconstruction for extensor mechanism repair^2^. However, no consensus exists on the optimal approach, and evidence in TKA patients remains limited. Using an autograft reduces the risk of infections and graft rejection when compared to allografts.

One study evaluating 18 knees with patellar tendon rupture post-TKA found that direct repair yielded variable outcomes, with only 25% showing favourable results. These findings support the need for augmentation to improve repair strength^3^.

Spoliti *et al* reported that autologous hamstring tendon grafts showed superior resilience and excellent outcomes in nine patients. However, in those with multiple knee surgeries, these grafts may be unavailable. Apart from donor site morbidity and pain in the thigh, there are chances of thigh muscle atrophy and increased graft incorporation time while using a hamstring graft. On the other hand, larger diameter peroneus grafts with greater strength and stability have the potential of faster rehabilitation and recovery at the cost of minimal donor site morbidity, such as ankle pain or weakness. Thus, peroneal tendon autografts present a viable alternative, offering reconstruction with an adequate tendon length and thickness^4^.

Maffulli *et al* assessed 254 patients who underwent allograft repair, reporting satisfactory clinical outcomes with careful surgical technique. Autografts are used in allograft restorations to minimise infection and autoimmune response risks^5^.

Our patient experienced traumatic patellar tendon rupture five days post-discharge. Intra-operative findings revealed insufficient soft tissue for direct repair, necessitating augmentation with the peroneus tendon. Post-operative outcomes were favourable, demonstrating good knee range of motion. This approach is recommended for acute traumatic patellar tendon disruption with poor intra-operative soft tissue.

In conclusion, early outcomes of patellar tendon reconstruction utilising an ipsilateral peroneus longus tendon autograft after knee arthroplasty are favourable. It facilitates early recovery, provides favourable clinical and functional outcomes, and reduces the risk of infection owing to stable graft fixation and lower the risk of autoimmune response in comparison to allograft constructs.
